# Rapid and non-invasive analysis of paracetamol overdose using paper arrow-mass spectrometry: a prospective observational study

**DOI:** 10.1186/s12916-024-03776-3

**Published:** 2024-11-25

**Authors:** Yufeng Zhou, Silothabo Dliso, Jennie Craske, Andrea Gill, Louise Bracken, Kiran Landa, Philip Arnold, Laura Walker, Ionela Grasim, Gabrielle Seddon, Tao Chen, Andrew S. Davison, Tung-Ting Sham, Barry Smith, Daniel B. Hawcutt, Simon Maher

**Affiliations:** 1 Department of Electrical Engineering and Electronics, University of Liverpool, Liverpool, L69 3GJ UK; 2 Alder Hey Children’s NHS Foundation Trust, Eaton Road, Liverpool, L12 2AP UK; 3 Department of Clinical Sciences, Liverpool School of Tropical Medicine, Liverpool, UK; 4 Department of Clinical Biochemistry and Metabolic Medicine, Liverpool Clinical Laboratories, Royal Liverpool University Hospitals NHS Foundation Trust, Liverpool, UK; 5 Institute of Life Course and Medical Sciences, University of Liverpool, Liverpool, L7 8TX UK; 6 NIHR Alder Hey Clinical Research Facility, Liverpool, L12 2AP UK

**Keywords:** Paracetamol, Saliva, Mass spectrometry, Paper arrow-mass spectrometry, Ambient ionisation

## Abstract

**Background:**

Paracetamol is the most consumed medicine globally. Its accessibility contributes to common overdose. Paracetamol overdose is responsible for > 50% of acute liver failure cases, making it the second most common reason for a liver transplant. Rapid quantitation of paracetamol is crucial to guide treatment of paracetamol overdose. Current tests require invasive sampling and relatively long turnaround times. Paper arrow-mass spectrometry (PA-MS) combines sample collection, extraction, separation, enrichment and ionisation onto a single paper strip, achieving rapid, accurate, cost-effective and eco-friendly analysis direct from raw human saliva.

**Methods:**

To validate PA-MS against an established test, 17 healthy adults were recruited. Samples were collected before and at 15, 30, 60, 120 and 240 min after ingesting 1 g of paracetamol. Plasma measured with an established clinical test served as the reference standard to validate PA-MS with three biofluids—plasma, resting saliva (RS) and stimulated saliva (SS). Participants’ views of blood, RS and SS sampling procedures were assessed qualitatively. Cross-validation was assessed using Lin’s concordance correlation coefficients (*CCC*), Bland–Altman difference plots, and ratios of PA-MS to the reference standard test.

**Results:**

PA-MS using stimulated saliva offers a reliable alternative to intravenous blood sampling. The *CCC* is 0.93, the mean difference with the reference test is − 0.14 mg/L, and the ratios compared to the reference test are 0.84–1.27 from correlated samples collected at 5 intervals over 4 h for each participant.

**Conclusions:**

Paracetamol detection from SS with PA-MS provides a reliable result that can aid timely treatment decisions. Differences between paracetamol concentration in resting and stimulated saliva were also identified for the first time, highlighting the importance of standardising saliva collection methods in general. This study marks a major milestone towards rapid and convenient saliva analysis.

**Supplementary Information:**

The online version contains supplementary material available at 10.1186/s12916-024-03776-3.

## Background

As an analgesic and antipyretic that patients can readily access over the counter (OTC), paracetamol (acetaminophen, APAP) consumption equates to about 6300 tonnes/year in the United Kingdom (UK) [1]. APAP accessibility, as an OTC drug, contributes to its common misuse and overdose (OD). In the UK alone, there are about 100,000 cases of APAP OD attending Emergency Departments every year, and around 50,000 hospital admissions are linked to resulting hepatotoxicity [2]. In the United States of America (USA), one in 20 emergency department visits was related to APAP OD [3]. APAP OD can induce lethal hepatic injury if not diagnosed and treated promptly [4], accounting for 56% (range 50.0–77.8%) of severe acute liver injury (ALI) or acute liver failure (ALF) [2]. Moreover, 29% of those who developed ALF due to APAP OD underwent a liver transplant, with a 28% mortality rate [5]. A literature review from 2017 to 2022 estimated worldwide APAP poisonings of 7.4 (range 0.1–63.6) per 100,000 population per year, which was close to liver cancer’s incidence of 9.3 per 100,000 population per year in 2020 [2, 6]. There is no doubt that APAP OD still represents a major global public health concern [2].

N-acetylcysteine (NAC), the antidote for APAP OD, can prevent most ALI if administered within 8 h of APAP ingestion, but it is only partly effective when given between 8 and 24 h and ineffective if given 24 h post-ingestion [7]. Delayed NAC treatment is clearly associated with poor outcomes, such as liver failure, transplant, and death, due to its time sensitivity [8]. Currently, treatment with NAC will commence only if paracetamol concentration is equal to or higher than the treatment line on the Rumack–Matthew nomogram [9]. This means, in most cases, treatment must wait for paracetamol concentration to be determined first. Currently, paracetamol is measured from a venous blood sample collected invasively by professionals. The analytical test is conducted in the central laboratory, and the turnaround time for results is 1 h at best but can be as long as 2–12 h [10]. Additionally, increasing waiting times in emergency departments can cause further testing and treatment delays, inhibiting NAC's effectiveness in preventing ALI. Therefore, swift and precise determination of paracetamol concentration is vital for timely clinical decision-making in cases of paracetamol OD [2]. A rapid and non-invasive approach with commensurate analytical performance will support clinicians in precisely and rapidly identifying patients at risk of hepatic injury and reduce the delivery time for antidote treatment, resulting in better outcomes and reduced costs [11].

To address this issue, we devised an innovative approach that seamlessly combines paper chromatography with mass spectrometry, termed paper arrow-mass spectrometry (PA-MS). The entire PA-MS process, from sample to result, is remarkably simple and can be readily completed in under 10 min, requiring only 2 µL of raw saliva. The approach exhibits superior analytical performance, surpassing that of liquid chromatography (LC)-MS [12]. PA-MS yields a linear response across the target toxic paracetamol concentration range, which includes 100 mg/L at 4 h post-ingestion of paracetamol and 15 mg/L at 15 h post-ingestion as defined by the Medicines and Healthcare products Regulatory Agency (MHRA), UK [13].

**Fig. 1:**
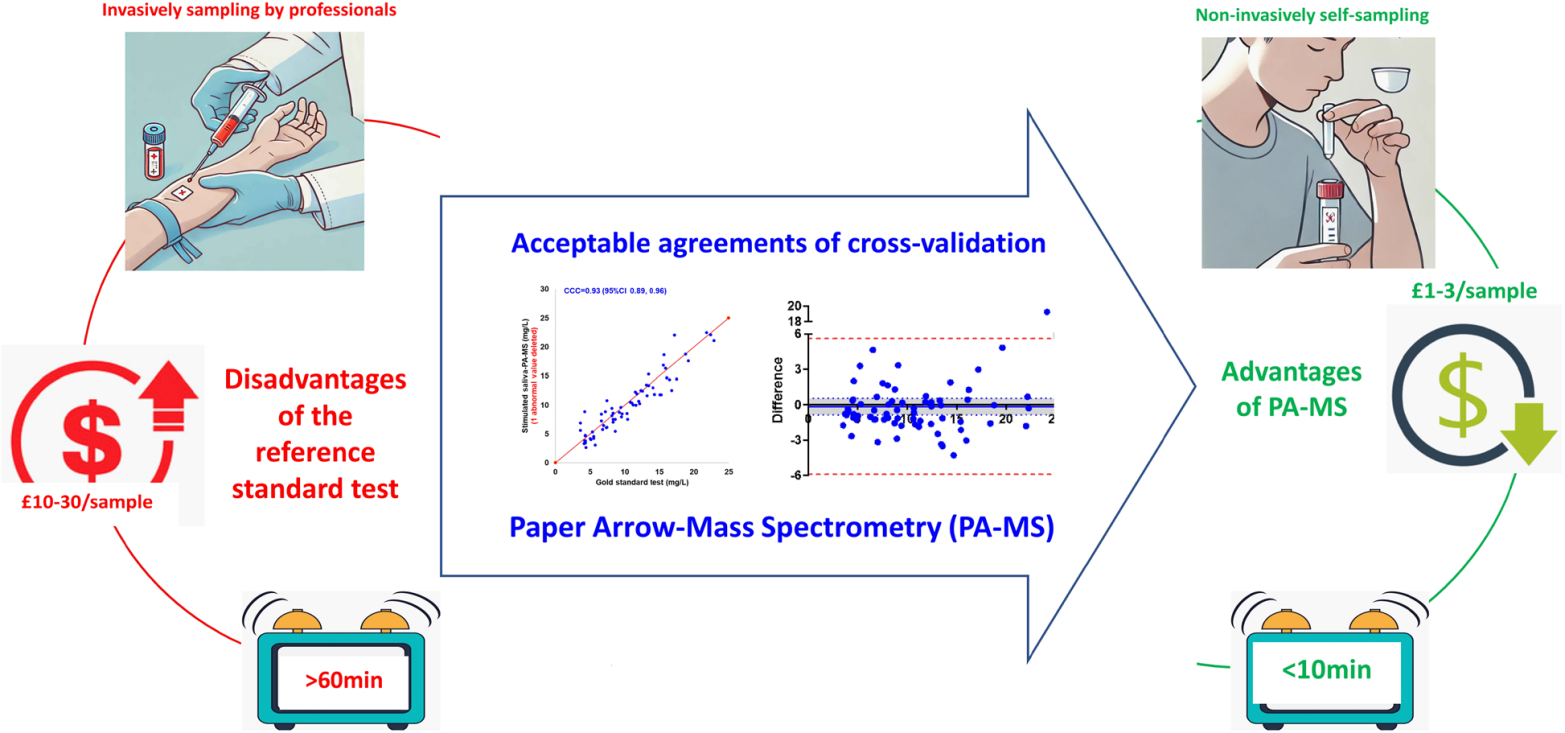
Successful validation of paper arrow-mass spectrometry (PA-MS), a paper-based technique used to test saliva samples, against an established (reference standard) test

In this study, PA-MS for paracetamol quantitation is rigorously cross-validated against an established clinical assay to determine its suitability for clinical use. It is also concerned with the effectiveness of salivary paracetamol as a reliable alternative and the acceptability of saliva sampling methods, in which PA-MS demonstrates advantages over the current test (Fig. 1).

## Methods

### Study design

This prospective observational study aimed to validate the efficacy of PA-MS against an established clinical assay for paracetamol quantitation. Plasma paracetamol detected with an automated enzymatic/colourimetric acetaminophen assay (Abbott Laboratories, Illinois, United States of America (USA)) served as the clinical reference standard assay. Paracetamol detected with PA-MS from three biofluids (plasma, RS, and SS) are considered to be three index tests; they were acronymised as plasma-PA-MS, RS-PA-MS and SS-PA-MS. By comparing the results of plasma-PA-MS with the reference standard assay, we sought to assess the reliability of PA-MS itself. Furthermore, the study involved a comparison of results from saliva samples analysed by PA-MS with those from the reference standard assay. This comparison aimed to determine whether RS and/or SS could serve as reliable alternatives to blood for detecting paracetamol concentrations (Supplemental Fig. 1).

### Participants recruitment

Eligible subjects were aged 18 years or older and had not taken any paracetamol 7 days prior to participation. Recruitment occurred at Alder Hey Children’s NHS Foundation Trust (referred to as Alder Hey Hospital) between April and June 2023. Participants with blood concentrations exceeding twice the upper limits of normal for alanine transaminase (ALT), aspartate transaminase (AST), alkaline phosphatase (ALP), and creatinine were excluded. Relevant tests were conducted during the baseline assessment. Participants who were allergic to paracetamol, pregnant or breastfeeding were also excluded. Written informed consent was obtained from all participants in the study, and participants were free to withdraw from the study at any time without providing a reason (ethical approval reference number: 11422).

### Sampling procedures

Blood samples were collected from a venous cannula into a Li-heparin 1.3-mL tube (Sarstedt, Nümbrecht, Germany). RS was collected passively by drooling for 2 min and spitting into a 5-mL sterile microcentrifuge tube. SS was collected by chewing a cotton swab (Salivette, Sarstedt, Nümbrecht, Germany) for 1 min. The blood samples, RS and SS, were collected simultaneously within 3 min. Baseline samples were collected before two tablets of 500 mg paracetamol were taken; further samples were collected at 15, 30, 60, 120, and 240 min after ingesting 1 g paracetamol. A light lunch was provided after the samples were collected at 120 min. The whole process is depicted in Additional file 1: Fig. S2.

**Fig. 2:**
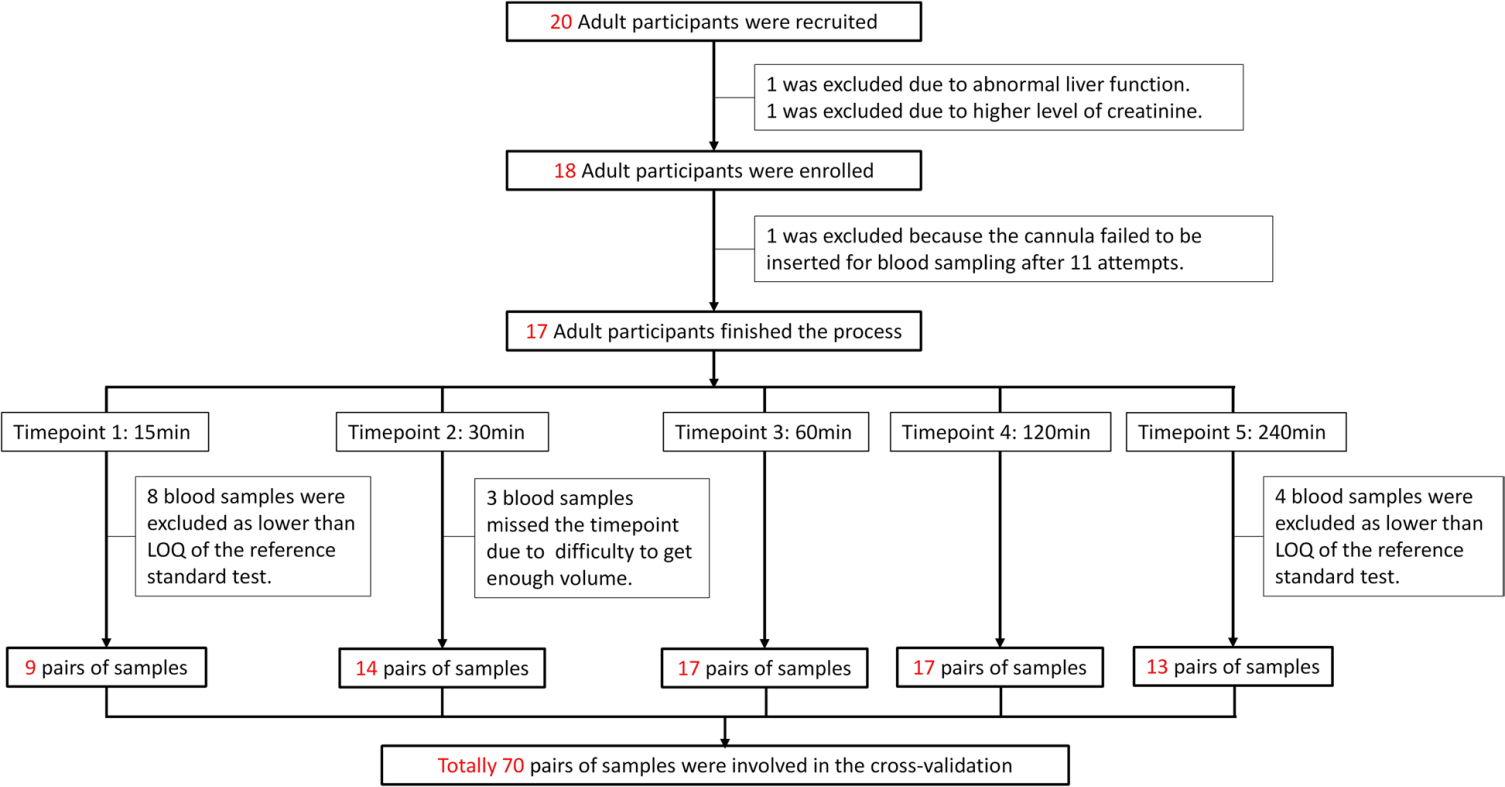
Flowchart of participants and samples involved in this study

### Reference standard test procedure

To proceed with the reference standard test procedure, 1.3 mL of blood was collected in a Li-heparin tube and then centrifuged for 5 min. The resulting supernatant plasma was analysed using an automated enzymatic colourimetric paracetamol assay (Abbott Laboratories, IL, USA) following standard protocols at Alder Hey Hospital, Liverpool (UK). The enzymatic reagents reacted with paracetamol, and the final reaction product, 4-(4-iminophenol)−2,5-dimethylcyclohexadiene-1-one, was measured at 660 nm using an Alinity ci-series instrument (Abbott Laboratories, Illinois, USA). Key analytical performance metrics for the reference test are noted in Additional file 1: Table S1.

### PA-MS procedure

PA-MS requires only 2 µL of raw sample applied directly to the paper arrow substrate and approximately 5 min for integrated paper chromatography separation without any sample pre-treatment prior to MS analysis [12]. Briefly, the absorbent substrate used is arrow-shaped Whatman grade 1 chromatography paper (Whatman, Maidstone, UK) with pre-loaded isotopically labelled internal standard (paracetamol-D4). The pre-test preparation involves adding a 2-µL sample onto the shaft of the arrow-shaped paper, which is then dried within 1 min. Next, the flat end of the shaft is dipped into a solvent mixture consisting of 50 mM ammonium formate in 9:1 ethyl acetate: formic acid (v/v). This solvent mixture carries paracetamol and separates it from the matrix, concentrating it at the arrowhead. This process takes 5 min. Finally, the analyte is physically isolated by cutting the arrowhead from the shaft for direct MS analysis. Paper arrow is amenable for testing with a wide variety of mass spectrometers; in this study, MS analysis was conducted predominantly with a Thermo Scientific Orbitrap Exploris 240 mass spectrometer (Thermo Fisher, Waltham, MA, USA). The MS analysis is conducted within 2 min. The mass spectrometer operational parameters have been reported previously [12].

Key analytical figures of merit for PA-MS are summarised in Additional file 1: Table S1. PA-MS analysis was performed on the same day as the reference standard test. The results of PA-MS were blind to the analyst of the reference standard test and vice versa.

### Participants’ views of the three sampling procedures

A semi-quantatitive, 5-point Likert questionnaire was developed to assess participants’ (dis)comfort, (in)convenience, and preference regarding blood, RS and SS sampling procedures. Open-ended questions were also included to collect information about any discomfort they experienced during the three sampling procedures. The short questionnaire (requiring 5–10 min to complete) was completed after the final samples were collected at 240 min.

### Further test of PA-MS on a portable mass spectrometer

We further conducted some initial tests with PA-MS on a portable mass spectrometer to highlight its suitability as a potential point-of-care test (POCT). Briefly, 20 μg/mL paracetamol-D4 and paracetamol at 0, 2.5, 5, 10, 20, 30, 50, 100 and 200 μg/mL were spiked into purified water and stimulated saliva separately. To conduct PA-MS, 4 μL of the spiked samples were applied onto paper arrow substrates. After 5 min paper chromatography, the arrowheads were cut off for analysis with a portable mass spectrometer (ACQUITY QDa, Waters, Wilmslow, UK) [14]. Calibration curves of purified water and saliva were constructed using the peak area ratio of paracetamol-D4 and paracetamol. Each concentration was carried out in triplicate.

### Statistical analysis and criteria of cross-validation

The detected paracetamol concentrations were described using the mean and standard deviation (SD). Correlation, agreement, and bias estimation tests were conducted with Lin’s concordance correlation coefficient (*CCC*) [15], Bland–Altman difference plots, and ratios of the index tests over the reference standard test. A one-way ANOVA was used to compare 5 different time points.

In clinical measurement, comparing a new measurement technique with an established one is needed to decipher whether they agree sufficiently for the new to replace the old. Such investigations can often be analysed inappropriately, notably by using correlation coefficients [16], which can be misleading [17, 18]. Instead, Lin’s concordance correlation coefficient (*CCC*) test addresses this limitation by combining measures of precision and accuracy to assess how well the data pairs fall along the line of perfect concordance [19]. Specifically, *CCC* evaluates both the correlation between the two methods (as Pearson does) and the proximity of the fitted line to the line of identity [19]. This makes CCC a robust measure of agreement suitable for method comparison studies that aim to ensure one method can reliably substitute the other [19]. Furthermore, this study conducted Bland–Altman difference plots to assess the agreement between PA-MS and the reference test by evaluating the difference between the methods across the range of measured values [17, 18]. This graphical method helps to identify any systematic bias and the presence of outliers. It also provides limits of agreement, indicating how well the two methods can be used interchangeably [17, 18].

The study also aimed to investigate the participants’ opinions and preferences regarding blood, RS, and SS sampling procedures. The data was presented in the form of mean rank, and the Kruskal–Wallis test was used to compare the results. Responses related to any discomfort experienced during the sampling processes were categorised in accordance with qualitative content analysis [20]. Statistical analysis was performed using the following software: IBM SPSS Statistics, version 25 (IBM Corp.), GraphPad Prism 8.0.2 (GraphPad Software, San Diego, USA), and Microsoft Excel. Statistical significance was set at *p* values < 0.05.

Following ICH guideline M10 on bioanalytical method validation and study sample analysis [21], the relevant validation criteria were determined according to published guidelines as summarised in Additional file 1: Table S2 [22, 23].

## Results

### Participants’ demographics and sample exclusion

In this study, 20 participants were initially recruited. However, two of them were later excluded due to abnormal liver or kidney function, and a further participant was excluded as we failed to obtain blood samples after multiple unsuccessful attempts. Consequently, 17 participants were enrolled and completed the study. Among them were five males and 12 females, with 12 being of white ethnicity and five being non-white. The average age of the participants was 36.8 years, ranging from 19 to 55 years. Theoretically, there should be 17 × 5 = 85 pairs of samples. However, practical difficulties led to the omission of 3 blood samples because of missing the accurate time points. Another 12 samples were excluded because the reference standard test failed to report exact values of paracetamol concentration lower than 3 mg/L (the limit of quantitation of the reference standard test), even though the index tests of PA-MS can provide precise concentration values between 0.2 and 3 mg/L (Additional file 1: Table S1). Consequently, 15 pairs of samples were excluded due to the low sensitivity of the reference standard test. In total, 70 pairs of samples were involved in the cross-validation process (Fig. 2), exceeding the minimum requirement of 40 pairs of samples for method validation [22]. Additional file 1: Table S3 displays concentrations of paracetamol detected with the reference test and PA-MS for the three biofluids (plasma, RS, SS) over the five different time points.

### Results of Lin’s concordance correlation

**Fig. 3:**
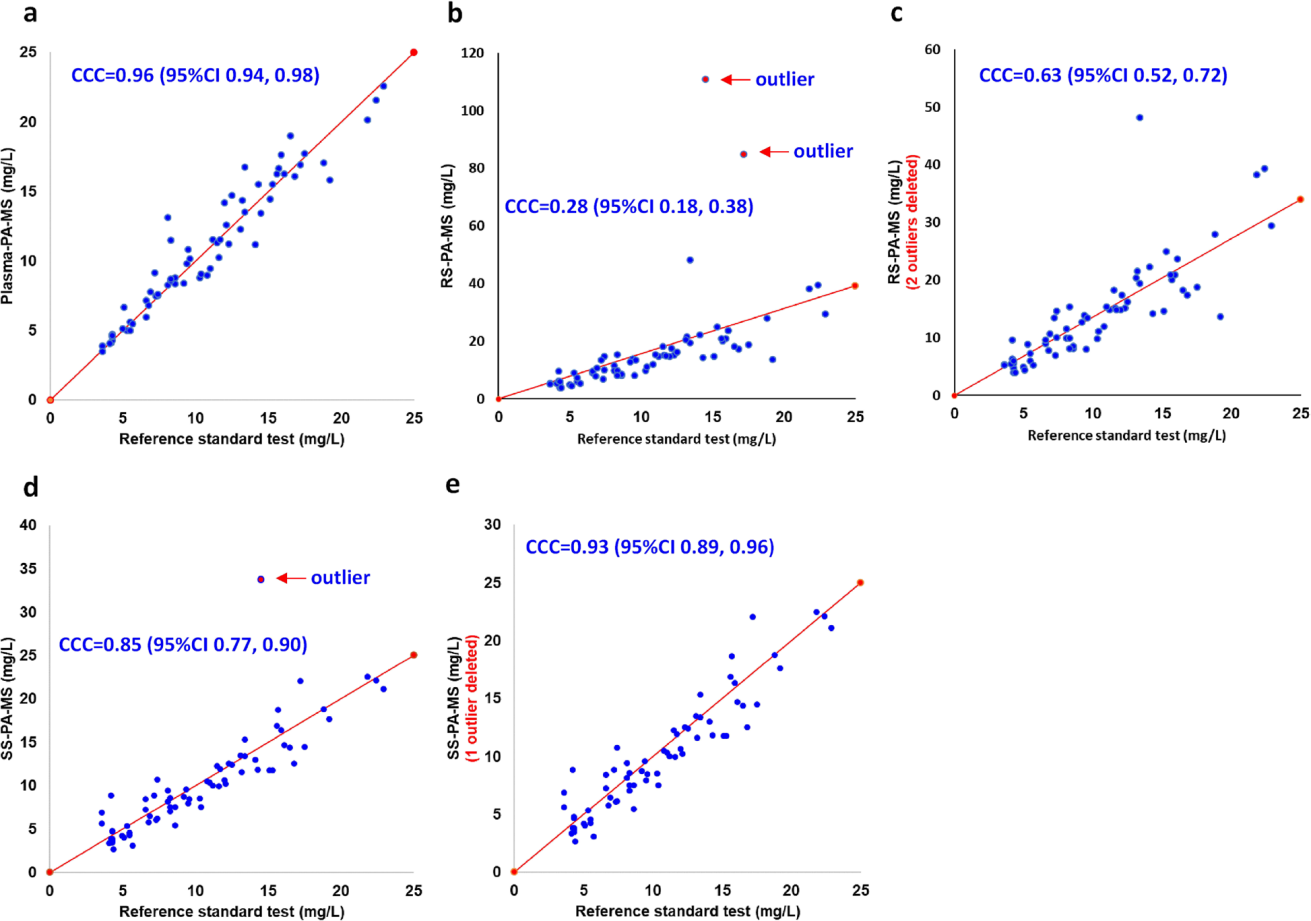
Lin’s concordance correlation of the three index tests compared with the reference standard test. **a** Correlation of plasma-PA-MS with the reference standard test. **b** Correlation of RS-PA-MS with the reference standard test. Two outliers are labelled (higher than *mean* ± 2*SD*). **c** Correlation of RS-PA-MS with the reference standard test (excluding two outliers). **d** Correlation of SS-PA-MS with the reference standard test. One outlier is labelled (higher than *mean* ± 2*SD*). **e** Correlation of SS-PA-MS with the reference standard test (excluding one outlier)

Paracetamol concentrations, as determined by PA-MS, exhibited a positive correlation compared to the reference standard method. Lin’s concordance correlation coefficients (*CCC*) are demonstrated in Fig. 3. Notably, Plasma-PA-MS exhibited the highest correlation with the reference standard test, yielding a *CCC* as high as 0.96 (Fig. 3a). On the other hand, RS-PA-MS exhibited the poorest correlation, registering a *CCC* of 0.63 even after excluding two outliers (prespecified criterium, mean ± 2SD) (Fig. 3c). For SS-PA-MS, the *CCC* was 0.93 when excluding an outlier (Fig. 3e).

### Results of Bland–Altman difference plots

Bland–Altman plots indicated the mean bias between the PA-MS index tests and the reference standard test. The range of differences between plasma-PA-MS, RS-PA-MS, SS-PA-MS and the reference standard test was − 3.37 to 5.04 mg/L, − 5.57 to 96.28 mg/L, and − 4.27 to 19.25 mg/L, respectively (Additional file 1: Table S4).

**Fig. 4:**
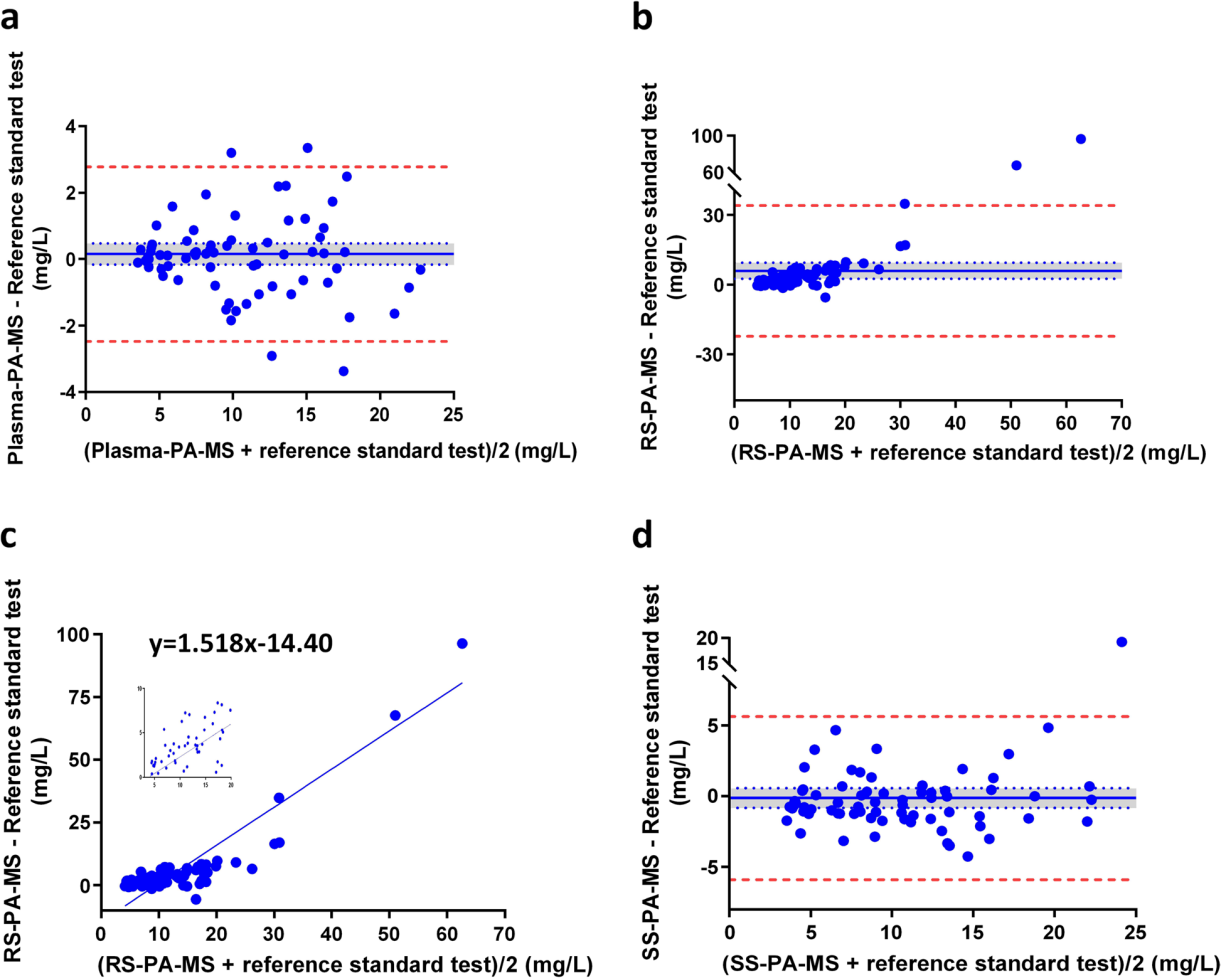
Bland–Altman difference plots between each of the three index tests and the reference standard test. **a** plasma-PA-MS. **b** RS-PA-MS. **c** Linear regression analysis between differences and means of RS-PA-MS and the reference standard test. **d** SS-PA-MS. Note: Solid blue lines indicate the means of differences between the index test and reference standard test; dotted blue lines indicate 95% CI (mean ± 1.96*SD*/√*N*); the blue areas indicate the ranges of 95% CI; and dashed red lines indicate 95% limits of agreement (mean ± 1.96SD)

In Fig. 4a, the mean of differences between plasma-PA-MS and the reference standard test was 0.15 mg/L (95% *confidence interval *(*CI*): − 0.17, 0.47), and no systematic error was observed between the two sets. The lower 95% limit of agreement was − 2.48 mg/L, and the upper limit was 2.78 mg/L. Notably, 65 out of 70 pairs fell within the 95% limits of agreement.

In Fig. 4b, the mean of differences between RS-PA-MS and the reference standard test was 5.93 mg/L (95% *CI*: 2.51, 9.34), implying a systematic error between the two datasets. The lower and upper 95% limits of agreement were − 22.21 mg/L and 34.07 mg/L, and 67 out of 70 pairs were within this range. Figure 4c shows the linear regression equation depicting the relationship between differences and means of RS-PA-MS and the reference standard.

For SS-PA-MS, as illustrated in Fig. 4d, the mean of differences was − 0.14 mg/L (95%*CI*: − 0.84, 0.56), revealing no systematic error between the two datasets. The lower and upper 95% limits of agreement were − 5.91 mg/L and 5.62 mg/L, with 69 out of 70 pairs falling within the 95% limit of agreement.

### Time curves of ratios of the three index tests of PA-MS over the reference standard test

**Fig. 5:**
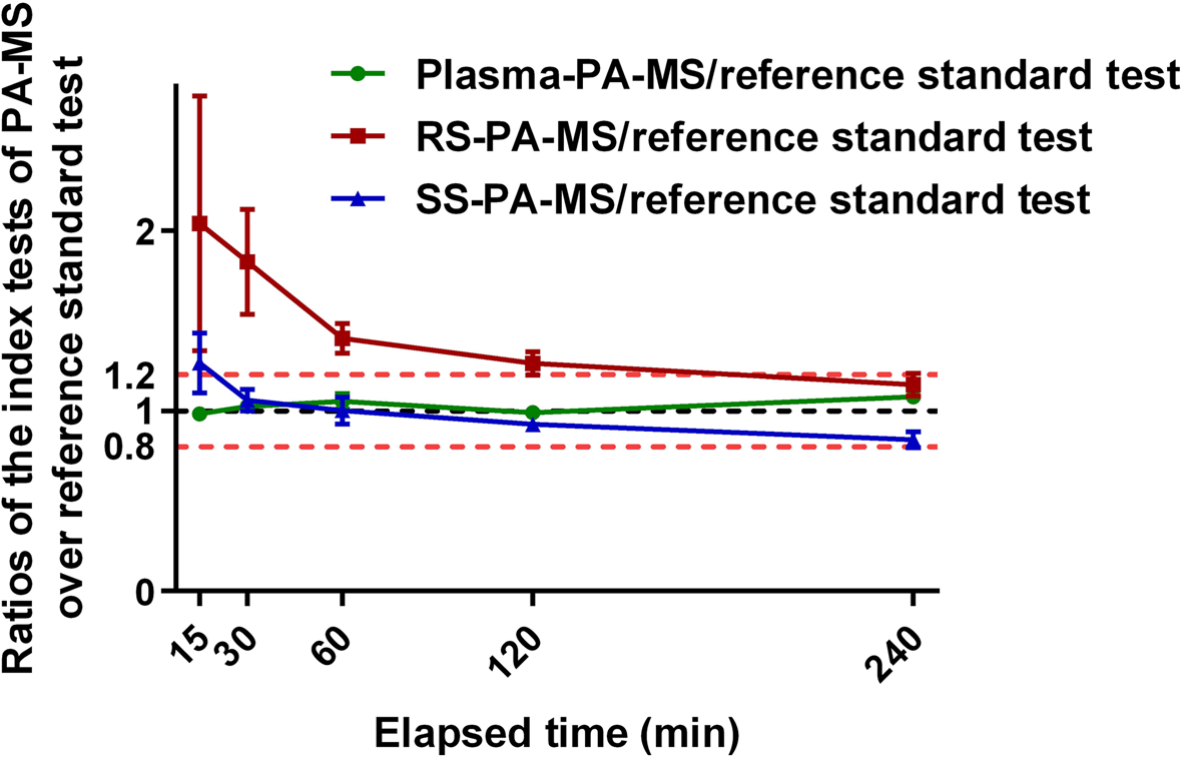
Time curves of ratios of the index tests of PA-MS over the reference standard test

Ratios of the index tests over the reference standard were plotted at 5 time points, over 15–240 min post paracetamol ingestion (Fig. 5). Ratios of plasma-PA-MS over the reference standard test (plasma-PA-MS/reference standard test) were between 0.98 and 1.08 at 5 time points with a mean of 1.025. 63 out of 70 pairs (90%) were within 1.0 ± 0.2. One-way ANOVA showed no statistically significant differences among all time points (*F* = 1.24, *p* = 0.30).

The ratios of RS-PA-MS over the reference standard test (RS-PA-MS/reference standard test) were between 1.15 and 2.04 (mean: 1.53). Only 22 out of 70 pairs (31.4%) were within 1.0 ± 0.2. Differences in RS-PA-MS/reference standard ratios among all time points were not statistically significant (*F* = 2.01, *p* = 0.10).

The ratios of SS measured by PA-MS over the reference standard test (SS-PA-MS/reference standard) were between 0.84 and 1.27 (mean: 1.02). 47 out of 70 pairs (67.1%) were within 1.0 ± 0.2. However, differences in SS-PA-MS/reference standard ratios among all time points were statistically significant (*F* = 3.96, *p* = 0.01).

### Qualitative data of participants’ views regarding the three sampling procedures

Mean rank was used to describe participants’ anxiety, discomfort, and convenience during blood, RS, and SS collection, and the Kruskal–Wallis test was used to compare them (Table 1). Though the differences were not statistically significant, the trend was clear: participants did not prefer blood sampling. The discomfort that participants experienced is categorised in Table 2 with original quotations noted.

**Table 1:** Rank of participants’ feelings and preferences regarding the three sampling procedures

Concerns	Sampling procedures	*n*	Mean rank	Explanation	Kruskal–Wallis *H* value	*p* value
Anxiety	RS	17	24.50	Middle	5.84	0.05
	SS	16	29.97	Least anxiety		
	Blood	15	18.67	Most anxiety		
Discomfort	RS	17	28.50	Least discomfort	4.48	0.11
	SS	16	25.56	Middle		
	Blood	15	18.83	Most discomfort		
Convenience	RS	17	23.47	Middle	4.93	0.09
	SS	16	20.13	Most convenience		
	Blood	15	30.33	Least convenience		
Preference	RS	16	24.88	Middle	2.22	0.33
	SS	16	19.78	Preferred mostly		
	Blood	14	26.18	Preferred least

**Table 2:** Categorising the discomfort experienced during sampling

Sampling	Categories	Original quotations
RS	Sore throat	My throat got sore without swallowing down saliva for 2 min
	Slow flow rate of saliva	I struggled to provide enough saliva
SS	Dryness of the cotton swab	The dry cotton in the mouth is not pleasant at all
		The dryness needs to be solved but no idea how to
	Unpleasant taste of the swab	Chewing the swab left an unpleasant taste in my mouth
		Chewing swab could be better tasting. Add pleasant flavours
		The cotton swab has a strong taste, more unpleasant than a 22G needle
Blood	Pain	The cannula caused a little bit of pain during the process
		Some pain but expected
	Difficult to access veins^a^	It took more than 15 min to get access to my vein
	Wasted blood and time	Cannula sampling took too long time to get enough blood to discard

^a^In this study, it took between 3 and 11 attempts for ten of the 18 participants (~ 55%) before blood samples were collected successfully

### PA-MS performance with a portable mass spectrometer

As shown in Supplemental Fig. 3, the LOQ of SS-PA-MS on a portable mass spectrometer was close to that of purified water, showing it can cover the target clinical concentration range with good linearity (*r*^2^ = 0.9983) and a LOQ of 7.2 mg/L. This test was performed without optimisation to demonstrate that PA-MS is compatible with other mass spectrometers, including those that can be considered as portable. 

## Discussion

As shown in the results, concentrations of paracetamol in plasma measured by PA-MS demonstrated excellent agreement with the reference standard test, with *CCC* as high as 0.96 and the mean of the difference between plasma-PA-MS and the reference standard test as low as 0.15 mg/L (95%*CI*: − 0.17, 0.47). All the cross-validation criteria (Additional file 1: Table S2) were met, indicating that PA-MS is a reliable method to detect paracetamol.

The novel PA-MS method evaluated herein seamlessly combines raw sample collection, extraction, enrichment, separation, and ionisation onto a single piece of paper. The entire process, from sample to result, can be readily completed in under 10 min, with a LOQ for plasma paracetamol as low as 0.21 mg/L, with excellent linearity across the range of 0.2–200 mg/L, requiring only 2 µL of sample volume [12]. For context, in a prior investigation, we detected paracetamol in saliva by LC–MS, and the sample preparation process took more than 1 h [14]. PA-MS shows great potential for clinical analysis in general.

RS-PA-MS showed poor correlation with the reference standard test, with a *CCC* of 0.63 even after excluding two outliers (Fig. 3c), much lower than the pre-set validation criterium of 0.85 (Additional file 1: Table S2). The Bland–Altman plot indicated a systematic error of 5.93 mg/L between RS-PA-MS and the reference standard test. Also, Fig. 4c showed a positive correlation between the differences and means of RS detected by PA-MS and the reference standard test, suggesting that the larger the mean of the two measurements, the larger the difference between them. Furthermore, ratios of RS-PA-MS/reference standard test were between 1.15 and 2.04 (mean: 1.53), and only 22 out of 70 pairs (31.4%) were within 1.0 ± 0.2. Using RS as the analysis media failed to reach most of the cross-validation criteria (Additional file 1: Table S2), exhibiting poor agreement with the reference standard test.

Paracetamol is a weak acid with pKa≈9.5. Thus, at physiological pH, it is almost entirely neutral [24]. With high permeability and a high fraction unbound to plasma proteins, paracetamol can be easily and rapidly transferred from blood into saliva along a concentration gradient [25, 26]. Primary saliva is secreted from the acini of the saliva glands. When the initial saliva flows through the ducts of saliva glands, sodium chloride, water and other electrolytes are reabsorbed by duct cells. Supposing paracetamol cannot be reabsorbed [27], it will be concentrated somewhat after travelling through the ductal system. This can explain why paracetamol concentration in resting saliva is higher than in blood. However, measuring paracetamol in resting saliva was deemed to be unreliable due to the poor agreement of RS-PA-MS with the reference standard test. It is necessary to use a larger population to fully investigate the relationship between paracetamol concentration in resting saliva and plasma due to the high inter-individual variation of RS-PA-MS. This is indicated by the high standard deviations (*SD*) of RS-PA-MS in Additional file 1: Table S3.

In contrast to RS, the *CCC* of stimulated saliva, SS-PA-MS, with the reference standard test was 0.93 when excluding an outlier (Fig. 3e). The mean of differences between SS-PA-MS and the reference standard test was − 0.14 mg/L. SS-PA-MS/reference standard test ratios were between 0.84 and 1.27 (mean: 1.02), and 47 out of 70 pairs (67.1%) were within 1.0 ± 0.2. These results fulfilled most of the cross-validation requirements, demonstrating that SS-PA-MS has a better agreement with the reference standard test than RS and suggesting SS is preferred over RS as a non-invasive alternative to blood for paracetamol detection.

Furthermore, Fig. 5 demonstrates that paracetamol concentrations in SS were consistently lower than RS by about one-third. To our knowledge, this is the first report of different paracetamol concentrations in stimulated and resting saliva. This finding affirms the need to establish and standardise saliva collection methods. From the literature, the earliest report exploring salivary paracetamol concentration did not mention the saliva collection method [28]. Several other studies have also not reported saliva collection methods either [29, 30], and some researchers incorrectly reported stimulated saliva from chewing as resting saliva [31]. Considering the differences between paracetamol concentrations in RS and SS we found in this study, those results from previous studies may require clarification regarding the sample collection method, highlighting the importance of a standardised saliva collection procedure. More importantly, it is reasonable to deduce that when analysing any molecular constituents in saliva, a comparison between RS and SS should be conducted routinely.

User-centred design (UCD) is an approach to design that prioritises users’ needs and preferences during the development process of products or systems. UCD is particularly important in the development of medical devices, as these devices directly impact patients’ health and well-being [32]. So far, few studies have explored personal experiences of sampling procedures [33]. Using a questionnaire, we adopted UCD principles and investigated participants’ views and preferences regarding blood, RS, and SS sampling procedures.

In this study, venous blood samples were collected invasively with a cannula. Ten out of 18 participants (~ 55%) endured more than three attempts of intravenous cannulation, so unsurprisingly, the blood sample collection procedure caused the most anxiety and discomfort and was the least convenient; participants ranked it the least preferable method (Table 1). These results highlight the need to develop non-invasive sampling methods.

Passive drool was used to collect RS, which the participants ranked as causing the least discomfort. On the other hand, chewing cotton swabs to collect SS was preferred by most of the participants as it was more convenient and less stressful (Table 1). However, the discomfort caused by SS ranked higher than RS. Based on Table 2 descriptions, discomfort caused by SS sampling fell into two categories: unpleasant taste and dryness of the cotton swab. These comments identified existing drawbacks in collecting stimulated saliva. This meaningful feedback will guide future research to optimise the procedure for stimulated saliva sampling, for example, adding some pleasant flavours to the collection substrate, as one of the participants suggested.

Besides the acceptable agreement with the reference standard test and the positive feedback from participants, SS-PA-MS also demonstrated several practical advantages over the reference standard test. The practical features of the two approaches are summarised in Additional file 1: Tables S5 and S6. SS-PA-MS was quicker, requiring fewer consumables and less clinician contact time, making it a compelling alternative to the current reference standard test.

Whilst PA-MS offers precise quantitation in keeping with stringent clinical analysis requirements, it also exhibits other important advantages such as speed of analysis, simplicity and ease of testing, low cost, and environmental friendliness. As a paper-based technique, the only consumables are the paper substrate and a few µL of solvent. Moreover, the promising performance of PA-MS lays a solid foundation for developing it into a POCT. To do so, PA-MS needs to be coupled with a portable mass spectrometer. In a preliminary experiment, we demonstrated this potential, showing good linearity within the target range of toxic concentrations using a portable mass spectrometer (Supplemental Fig. 3). This is the subject of ongoing research to develop a one-step automated process that can be seamlessly administered by healthcare professionals.

### Limitations

Three limitations of this study are noted. First, saliva was mechanically stimulated by chewing cotton swabs. Other stimulation methods, like facial vibrotactile or chemical citric acid stimulation, deserve exploration. Second, the participants in this study were all healthy adults. Other types of populations should be involved in future studies. Last, possible endogenous and exogenous interferences in SS detected by PA-MS should be tested further. We are actively engaged in follow-up research studies to investigate these.

### Conclusions and further studies

PA-MS is demonstrated to be a reliable method to detect paracetamol in plasma. Differences between paracetamol concentrations in resting saliva and stimulated saliva were also identified for the first time, highlighting the importance of establishing and standardising collection methods for salivary analysis. Whilst resting saliva is excluded as a reliable biofluid to detect paracetamol, stimulated saliva detected by PA-MS is reliable, offering a non-invasive, low-cost, sensitive and convenient method for paracetamol quantitation, requiring only 2 µL of raw sample and < 10 min turnaround time for results. It is a promising candidate to be introduced into routine clinical practice and as a potential POCT for paracetamol overdose, enabling informed treatment decisions beyond traditional settings (e.g. in prisons, community pharmacies and general practitioner surgeries), which can lead to reduced hospitalisation time, better cost-effectiveness and improved patient outcomes.

This study marks a major milestone towards rapid and convenient saliva analysis. As demonstrated with paracetamol detection, PA-MS can avail new clinical research avenues in relation to personalised medicine. Due to its universality, PA-MS can be used for a wide range of biochemical testing. For instance, in other ongoing studies, we have applied PA-MS to the measurement of other analytes of clinical relevance (such as cortisol, cortisone, tacrolimus, and tenofovir) in a range of biofluids (e.g. whole blood, urine, and sweat). This study, which focused on paracetamol measurement from saliva and blood, demonstrated that PA-MS is a reliable technique according to clinical guidelines. Future work will avail the universal sensing capability of PA-MS for the analysis of a wide range of medicines and markers of interest within broader clinical and societal scenarios.

## Supplementary Information

Additional file 1: Table S1 Analytical performance of the index tests of PA-MS and the reference standard test. Table S2 Summary of the cross-validation criteria. Table S3 Paracetamol concentrations (mg/L) measured using the three index tests of PA-MS and the reference standard test. Table S4 Frequency of the differences between the index tests and the reference standard test (percentage out of 70 pairs). Table S5 Comparison of sampling procedures between SS-PA-MS and the reference standard test. Table S6 Comparison of analytical procedures between PA-MS and the reference standard test. Figure S1 Schematic illustrating the cross-validation process. Figure S2 Timeline of the study process. Figure S3 Calibration curves measured with PA-MS on a portable mass spectrometer.

## Data Availability

Data is provided within the manuscript or supplementary information file. The original datasets used and/or analysed during the current study are available from the corresponding author upon reasonable request.

## Abbreviations

PA-MS: Paper arrow-mass spectrometry
RS: Resting saliva
SS: Stimulated saliva
*CCC*: Lin’s concordance correlation coefficient
OTC: Over the counter
APAP: Acetaminophen
UK: United Kingdom
OD: Overdose
USA: United States of America
ALI: Acute liver injury
ALF: Acute liver failure
NAC: N-acetylcysteine
LC-MS: Liquid chromatography-mass spectrometry
POCT: Point-of-care test
MHRA: Medicines & healthcare products regulatory agency
ALT: Alanine transaminase
AST: Aspartate transaminase
ALP: Alkaline phosphatase
ICH: The international council for harmonisation of technical requirements for pharmaceuticals for human use
LOQ: Limit of quantitation
*CI*: Confidence interval
*SD*: Standard deviation
UCD: User-centred design
