# Supplementary material for: Rapid and non-invasive analysis of paracetamol overdose using paper arrow-mass spectrometry: a prospective observational study
Source: BMC Med. 2024 Nov 25;22:553. doi: 10.1186/s12916-024-03776-3 (PMC11590362; doi:10.1186/s12916-024-03776-3)
Supplement: Supplementary file 1 — Additional file 1: Table S1 Analytical performance of the index tests of PA-MS and the reference standard test. Table S2 Summary of the cross-validation criteria. Table S3 Paracetamol concentrations (mg/L) measured using the three index tests of PA-MS and the reference standard test. Table S4 Frequency of the differences between the index tests and the reference standard test (percentage out of 70 pairs). Table S5 Comparison of sampling procedures between SS-PA-MS and the reference standard test. Table S6 Comparison of analytical procedures between PA-MS and the reference standard test. Figure S1 Schematic illustrating the cross-validation process. Figure S2 Timeline of the study process. Figure S3 Calibration curves measured with PA-MS on a portable mass spectrometer. [file 12916_2024_3776_MOESM1_ESM.docx]

**Additional File**

Table of Contents

[**Table S1 Analytical performance of the index tests of PA-MS and the reference standard test** 2](#_Toc182857394)

[**Table S2 Summary of the cross-validation criteria** 3](#_Toc182857395)

[**Table S3 Paracetamol concentrations (mg/L) measured using the three index tests of PA-MS and the reference standard test** 4](#_Toc182857396)

[**Table S4 Frequency of the differences between the index tests and the reference standard test (percentage out of 70 pairs)** 5](#_Toc182857397)

[**Table S5 Comparison of sampling procedures between SS-PA-MS and the reference standard test** 6](#_Toc182857398)

[**Table S6 Comparison of analytical procedures between PA-MS and the reference standard test** 7](#_Toc182857399)

[**Figure S1 Schematic illustrating the cross-validation process** 8](#_Toc182857400)

[**Figure S2 Timeline of the study process** 9](#_Toc182857401)

[**Figure S3 Paracetamol calibration curves measured with PA-MS on a portable mass spectrometer** 10](#_Toc182857402)

# **Table S1 Analytical performance of the index tests of PA-MS and the reference standard test**

| **Tests** | **LOD (mg/L)** | **LOQ (mg/L)** | **Concentration Range (mg/L)** | **Precision (%)** |
| --- | --- | --- | --- | --- |
| Reference standard test | 1.00^a^ | 3.00^a^ | 3.00-337.00^a^ | 0.6-4.6^b^ |
| Plasma-PA-MS^c^ | 0.07 | 0.21 | 0.20-200.00 | 1.1-6.0 |
| RS-PA-MS^c^ | 0.06 | 0.18 | 0.20-20000. | 0.7-4.2 |
| SS-PA-MS^c^ | 0.04 | 0.13 | 0.20-200.00 | 0.7-3.5 |

a Published in the instructions of the reference standard test kit;

b Within batch precision tests at 3 concentration levels of 9 mg/L, 67 mg/L, and 205 mg/L were carried out in the clinical laboratory of Alder Hey Hospital (N=15);

c Calibration curves were carried out at the University of Liverpool. Limit of Detection (LOD)=3.3×*SD* of Response/Slope; Limit of Quantification (LOQ) =10×SD of Response/Slope; Inter-assay precision was performed at 9 concentration levels from 0.2-200 mg/L.

Abbreviations: PA-MS: Paper arrow-mass spectrometry; RS: Resting saliva; SS: Stimulated saliva.

# **Table S2 Summary of the cross-validation criteria**

| **Statistical analysis** | **Parameters** | **Criteria** |
| --- | --- | --- |
| Lin’s concordance correlation coefficient | CCC (r) | >0.85 |
| Bland-Altman plot | 95% confidence interval (CI) of the mean of differences | Including 0 |
|  | 95% limits of agreement | Within ±10 mg/L^a^ |
|  | Correlation between differences and means | No correlation exists |
| Time curve of ratios of index tests of PA-MS / reference standard test | Ratios of the two tests’ results | 67% ratios are within 1.0±0.2 |
|  | Means of ratios at all time points | Within 1.0±0.2 |
|  | One-way ANOVA between 5 time points | *p* value >0.05 |

a. Limits of agreements were defined as 10% of 100 mg/L, as all patients with a timed plasma paracetamol level on or above 100 mg/L at 4 hours after ingestion should receive acetylcysteine. This is based on the treatment nomogram published by the UK’s Medicines and Healthcare products Regulatory Agency (MHRA) (https://www.gov.uk/drug-safety-update/treating-paracetamol-overdose-with-intravenous-acetylcysteine-new-guidance).

# **Table S3 Paracetamol concentrations (mg/L) measured using the three index tests of PA-MS and the reference standard test**

| **Timepoints (min)** | ***n*** | **Reference standard test** | **Plasma-PA-MS** | **RS-PA-MS** | **SS-PA-MS** |
| --- | --- | --- | --- | --- | --- |
| 15 | 9 | 13.97±5.24 | 13.52±4.79 | 28.59±32.05 | 16.73±7.96 |
| 30 | 14 | 14.37±5.69 | 14.60±5.42 | 26.48±20.27 | 14.47±5.26 |
| 60 | 17 | 11.26±3.21 | 11.67±3.14 | 15.45±5.13 | 10.62±2.16 |
| 120 | 17 | 8.56±2.87 | 8.47±3.21 | 10.58±3.41 | 7.77±2.46 |
| 240 | 13 | 5.10±1.24 | 5.56±1.98 | 5.75±1.35 | 4.18±1.28 |

# **Table S4 Frequency of the differences between the index tests and the reference standard test (percentage out of 70 pairs)**

| **Difference** | **Plasma-PA-MS minus reference standard test** | **RS-PA-MS minus**  **reference standard test** | **SS-PA-MS minus**  **reference standard test** |
| --- | --- | --- | --- |
| -10 to -5 mg/L | - | 1 (1.4%) | - |
| -5 to 0 mg/L | 31 (44.3%) | 13 (18.6%) | 45 (64.3%) |
| 0 to 5 mg/L | 38 (54.3%) | 35 (50.0%) | 34(48.6%) |
| 5 to 10 mg/L | 1 (1.4%) | 16 (22.9%) | - |
| ≥10 mg/L | - | 5 (7.1%) | 1 (1.4%) |

# **Table S5 Comparison of sampling procedures between SS-PA-MS and the reference standard test**

| **Practical issues** | **SS-PA-MS** | **Blood collection** |
| --- | --- | --- |
| Sample to collect | 2 µL Saliva | 1.3 mL Blood |
| Sample collection | Non-invasive | Invasive |
| Supplies required for sample collection | One collection tube | Blood collection tube, tourniquet, disinfection wipe, needle/cannula, medical adhesive, syringe, gauze, plaster ^a^ |
| Sample collection time | ~1 min | ~5-15 min (depending on the difficulty of venous access) ^a^ |
| Location of collection | Bedside / anywhere | Usually, in the phlebotomy department |
| Clinical staff | Self-sampling | Professional to collect samples |
| Cost of sampling | ~£0.70 | ~£5.30/attempt ^a^ |

^a^ In this study, 10 out of 18 participants required 3-11 attempts to collect intravenous blood samples successfully. In those cases, the supplies, staffing resources and costs for sample collection were multiplied accordingly. The cost is estimated to be £15.90 for 3 attempts and £58.30 for 11 attempts.

# **Table S6 Comparison of analytical procedures between PA-MS and the reference standard test**

| **Parameters** | **PA-MS** | **Reference standard test** |
| --- | --- | --- |
| Volume of sample per test | 2 µL | 50 µL |
| Sample preparation time before test | 5 min | 5 min |
| Volume of solvent for sample preparation | 25 µL | - |
| Detection time | 1.6 min | 16 min |
| Volume of solvent for detection | 40 µL | 150 µL |
| Detection site | Central laboratory or in-clinic (portable system) | Central laboratory |
| Turnaround time ^a^ | - | 75 min ^b^ |

^a^ The reported time is from the central laboratory receiving samples to sending out the results; note well that the time required for sample collection and transfer are not included.

^b^ Of 5518 paracetamol tests from March 2023-24 at Royal Liverpool University Hospital, the median turnaround time was 75 minutes, with a 25th percentile of 50 minutes and 75th percentile of 5 hours and 42 minutes.


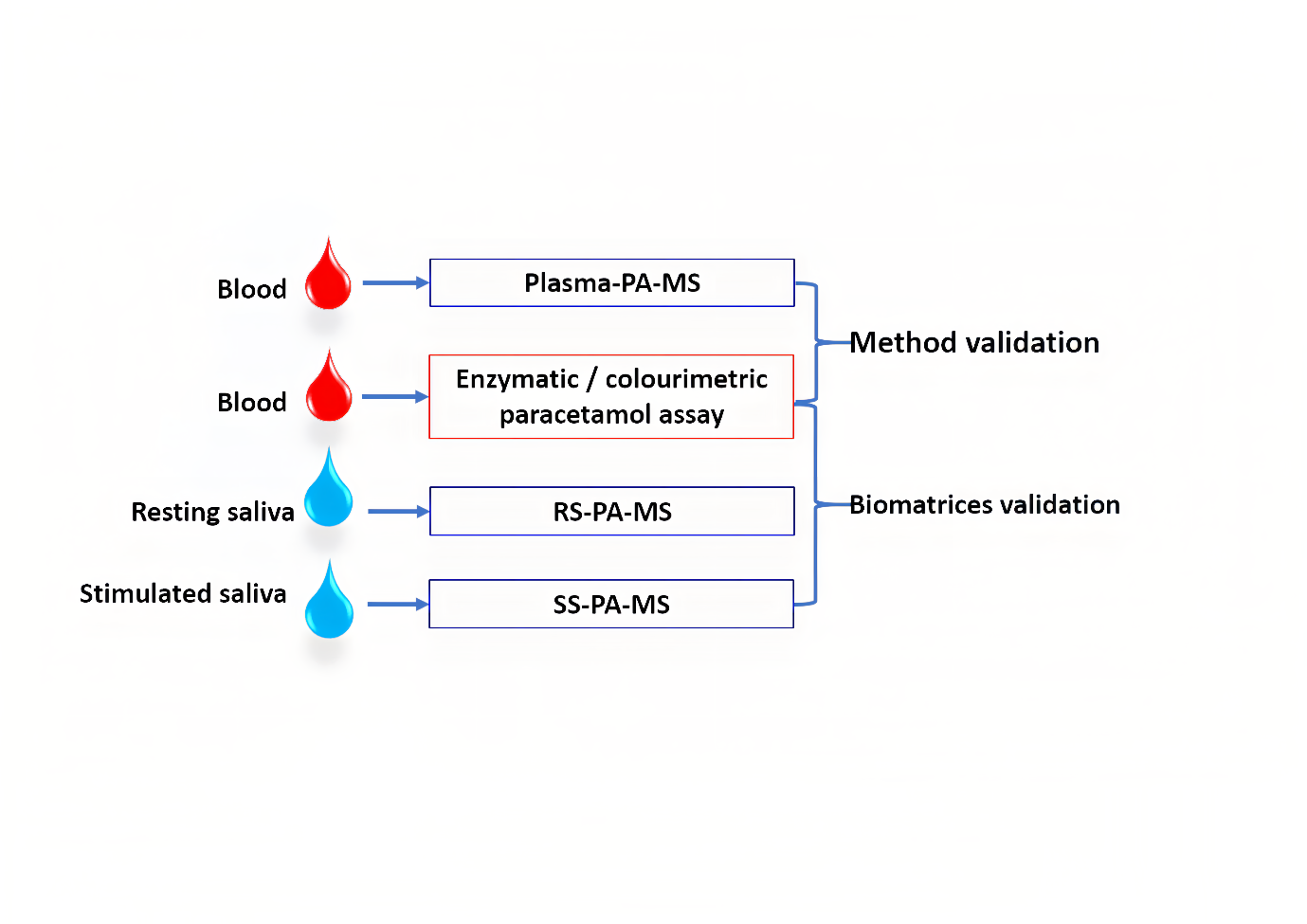


# **Figure S1 Schematic illustrating the cross-validation process**


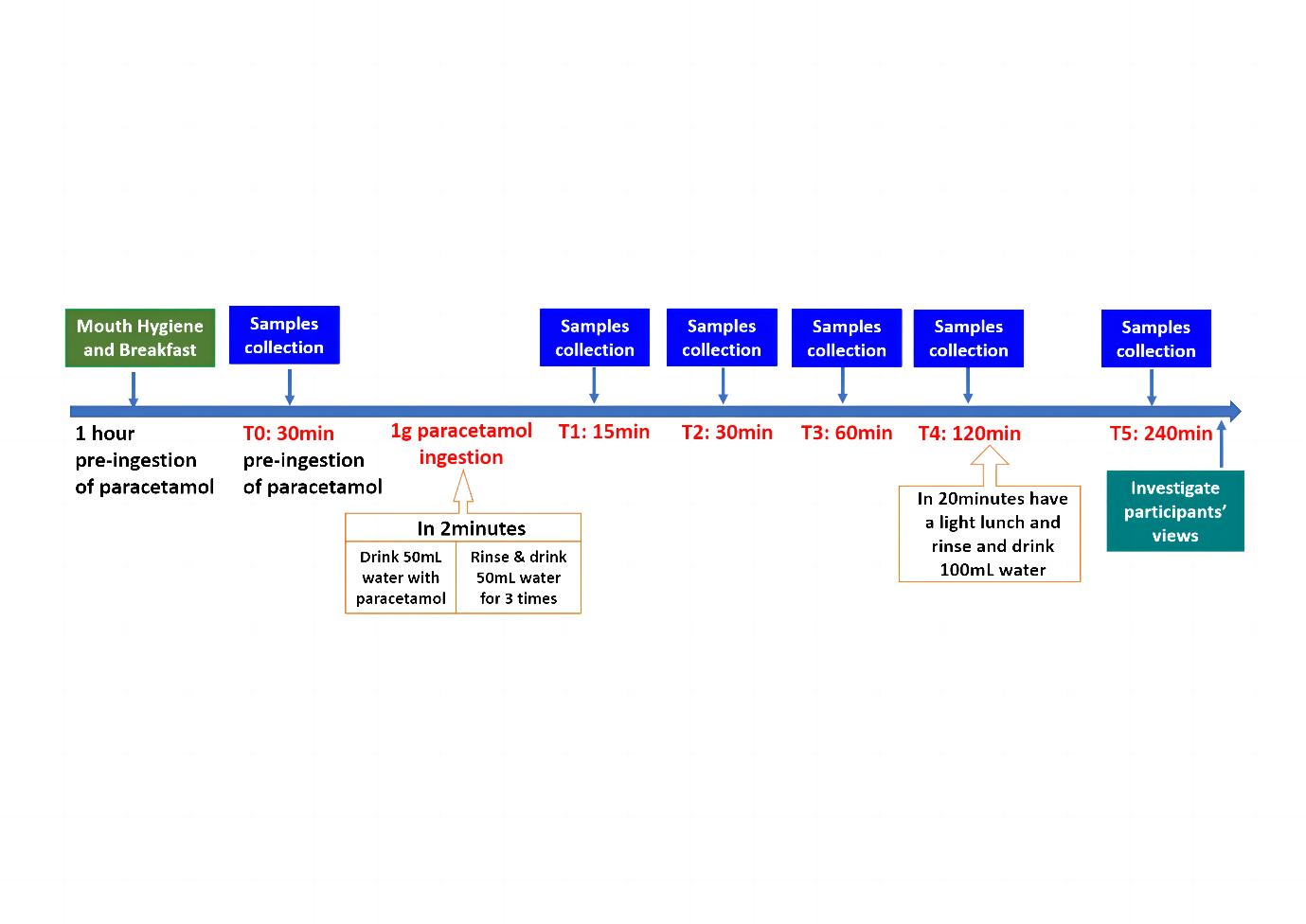


# **Figure S2 Timeline of the study process**


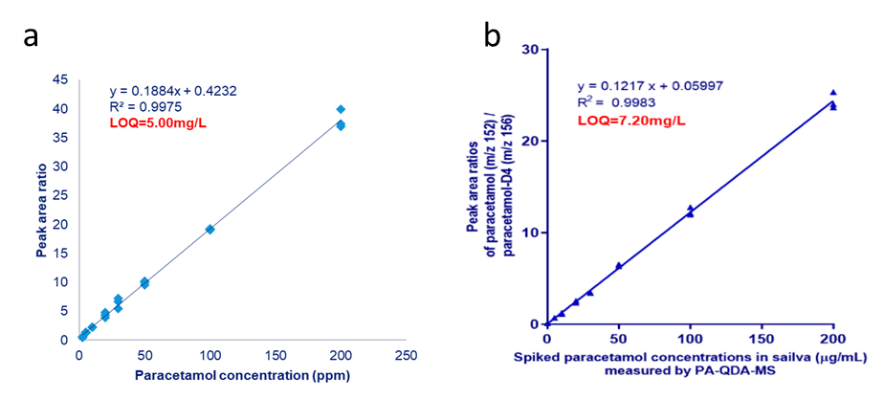


# **Figure S3 Paracetamol calibration curves measured with PA-MS on a portable mass spectrometer**

a Calibration curve of water samples, b Calibration curve of stimulated saliva samples.
